# Elevated transcription and glycosylation of B3GNT5 promotes breast cancer aggressiveness

**DOI:** 10.1186/s13046-022-02375-5

**Published:** 2022-05-07

**Authors:** Zhaorui Miao, Qianhua Cao, Ruocen Liao, Xingyu Chen, Xiaoli Li, Longchang Bai, Chenglong Ma, Xinyue Deng, Zhijun Dai, Jun Li, Chenfang Dong

**Affiliations:** 1 Department of Pathology and Pathophysiology, Department of Colorectal Surgery and Oncology, Key Laboratory of Cancer Prevention and Intervention, The Second Affiliated Hospital, Ministry of Education, Zhejiang University School of Medicine, 310058 Hangzhou, China; 2 Zhejiang Key Laboratory for Disease Proteomics, Zhejiang University School of Medicine, 310058 Hangzhou, China; 3 Abcam Plc, 1418-32 Moganshan Road, 311500 Hangzhou, China; 4 Department of Breast Surgery, First Affiliated Hospital, Zhejiang University School of Medicine, 310058 Hangzhou, China

**Keywords:** B3GNT5, Basal-like breast cancer (BLBC), SSEA-1, Cancer stem cells, Glycosylation

## Abstract

**Background:**

Basal-like breast cancer (BLBC) is the most aggressive subtype of breast cancer because of its aggressive biological characteristics and no effective targeted agents. However, the mechanism underlying its aggressive behavior remain poorly understood. β1,3-N-acetylglucosaminyltransferase V (B3GNT5) overexpression occurs specifically in BLBC. Here, we studied the possible molecular mechanisms of B3GBT5 promoting the aggressiveness of BLBC.

**Methods:**

The potential effects of B3GNT5 on breast cancer cells were tested by colony formation, mammosphere formation, cell proliferation assay, flow cytometry and Western blotting. The glycosylation patterns of B3GNT5 and associated functions were determined by Western blotting, quantitative real-time PCR and flow cytometry. The effect of B3GNT5 expression on BLBC was assessed by *in vitro* and *in vivo* tumorigenesis model.

**Results:**

In this study, we showed that B3GNT5 copy number amplification and hypomethylation of B3GNT5 promoter contributed to the overexpression of B3GNT5 in BLBC. Knockout of B3GNT5 strongly reduced surface expression of SSEA-1 and impeded cancer stem cell (CSC)-like properties of BLBC cells. Our results also showed that B3GNT5 protein was heavily N-glycosylated, which is critical for its protein stabilization. Clinically, elevated expression of B3GNT5 was correlated with high grade, large tumor size and poor survival, indicating poor prognosis of breast cancer patients.

**Conclusions:**

Our work uncovers the critical association of B3GNT5 overexpression and glycosylation with enhanced CSCs properties in BLBC. These findings suggest that B3GNT5 has the potential to become a prognostic marker and therapeutic target for BLBC.

**Supplementary information:**

The online version contains supplementary material available at 10.1186/s13046-022-02375-5.

## Background

β1,3-N-acetylglucosaminyltransferase (β3GnT) is a unique family of glycosyltransferase which attaches N-acetylglucosamine (GlcNAc) from UDP-GlcNAc to Gal on the non-reducing end of the carbohydrate chain with β1,3-linkage [1]. β1,3-N-acetylglucosaminyltransferase V (B3GNT5) is the fifth β3GnT enzyme to be verified as the most feasible candidate for synthesizing lactotriaosylceramide (GlcNAcβ1-3Galβ1-4Glcβ1-1Ceramide, Lc3Cer) [2]. As the sole enzyme for Lc3Cer synthesis, B3GNT5 plays a critical role in generation of lacto- and neolacto-series carbohydrate chains found on glycolipids, which are correlated with embryonic development and some malignant diseases [3–6]. The previous studies have demonstrated that Lc3Cer overexpression in multiple tumors is associated with poorer patient survival [7, 8]. A recent study have shown that augmented enzymatic activity of B3GNT5 resulted in accumulation of neolacto-series glycosphingolipids in tumor cells, impeding the immune surveillance [9].

The stage-specific embryonic antigen 1 (SSEA-1) regulated by B3GNT5 possesses a lacto-series carbohydrate structure which is considered as a potential biomarker of various tumors [10–12]. Intriguingly, accumulating studies have suggested that SSEA-1 is potentially associated with CSCs properties that have been shown to be highly tumorigenic [12–17]. In this study, we demonstrate that B3GNT5 expression is dramatically elevated in BLBC, a subtype that has poor clinical outcome due to its aggressiveness and no effective targeted agents [3]. B3GNT5 enhances SSEA-1 expression and CSCs properties of breast cancer cells through B3GNT5 overexpression and its glycosylation-mediated protein stabilization, promoting tumorigenesis.

## Methods

### Plasmids and antibodies

Human B3GNT5 gene was amplified from MDA-MB231 cDNA library, and sub-cloned into pLVX-puro and pLVX-neo, respectively. Antibody against FLAG was purchased from Sigma-Aldrich (catalog no. F3165). Antibody against GAPDH was purchased from Cell Signaling Technology (catalog no. 97,166). Antibody against mcherry was purchased from ABclonal (catalog no. AE002). CD44-APC (catalog no. 17-0441-81) and CD24-PE (catalog no. 12-0247-41) were purchased from Invitrogen. Antibody against SSEA-1 (catalog no. 125,602) and its IgM Isotype Ctrl antibody (catalog no. 401,602) was purchased from BioLegend. Goat Anti-Mouse IgM mu chain (Alexa Fluor® 488) was purchased from Abcam (catalog no. ab150121).

### CRISPR-Cas9 genome editing and verification

The design of gRNA targeting human B3GNT5 was carried out using online tools from Zhang’s lab [18], then the target sequence was cloned into lentiCRISPR v2 plasmid (Addgene). LentiCRISPR v2 plasmid was cotransfected into HEK293T with the packaging plasmid psPAX2 and pMD2.G using Lipofectamine™ 3000 (Thermofisher) for virus production. Filtered viral supernatants were used for transfecting MDA-MB231, BT549 and SUM159 cells. Cells were selected using puromycin (300ng/mL), and single cell was seeded into 96-wells flat-bottom plates (Corning). Cells from single-cell derived clones were harvested, and DNA was extracted for genome editing verification. The gRNA primers used for CRISPR-Cas9 were: 5’-CACCGCTCTTAAGCACACCTCAGCG-3’ (forward) and 5’-AAACCGCTGAGGTGTGCTTAAGAGC-3’ (reverse).

### Cell culture

All cells we used in this study were obtained from the American Type Culture Collection (Manassas, VA), where the cell lines were authenticated by STR profiling before distribution. MDA-MB231, SUM159, MCF7 and HEK293T cells were grown in Dulbecco’s modified Eagle’s Medium (DMEM)/F12 with 10% FBS. BT549 cells were grown in RPMI-1640 supplemented with 10% FBS. All the cells were cultured and stored according to the instruction from the ATCC. For establishing stable transfectants with overexpression of B3GNT5, MCF-7, MDA-MB231 and BT549 cells were transfected with pLVX-puro-B3GNT5-WT-Flag; MDA-MD231 KO and BT549 KO cells were transfected with pLVX-neo-B3GNT5-WT-Flag; stable clones were selected using 300 ng/mL puromycin and 400 µg/mL neomycin for 4 weeks, respectively.

### Quantitative real-time PCR

Total RNA was extracted from cells by AG RNAex Pro Reagent (Accurate Biology) according to the manufacturer’s instructions. Reverse transcription was performed with the Evo M-MLV II Reverse Transcriptase (Accurate Biology). Real-time quantitative PCR (RT-qPCR) was performed using SYBR Green Premix Pro Taq HS qPCR Kit (Accurate Biology) according to the manufacturer’s protocol. Gene expression level was normalized to GAPDH level in respective samples as an internal control, and the results were performed with at least three independent experiments. The primers used for RT-qPCR were: 5’-GGGCCTCGCTACCAATACTTG-3’ (forward) and 5’-CGGAACGTCGATCATAGTTTTCA-3’ (reverse) for B3GNT5; 5’-TGCACCACCAACTGCTTAGC-3’ (forward) and 5’-GGCATGGACTGTGGTCATGAG-3’ (reverse) for GAPDH.

### Colony formation assay

Colony formation assay was performed using double-layer soft agar in 24-well plates with a bottom layer of 0.7% agar and a top layer of 0.35% agar. Different cells were seeded in 24-well plates and cultured in desired medium with proper cell counts at 37 °C, and the colonies were counted after cultivation for 21 ~ 28 days.

### Mammosphere assay

Mammosphere assays were performed as previously described [19]. Briefly, by plating single-cell suspensions into ultralow-attachment 6-well plates (Corning) in mammosphere culturing conditions and counting after 14 d. In subsequent passage, single cell from the former generation spheres were seeded at the same concentration in ultralow-attachment 6-well plates. Spheres were counted 14 days after seeding.

### Flow cytometry analysis and fluorescence-activated cell sorting

In order to detect CD44^high^/CD24^low^ population, single-cell suspensions were counted and incubated in 100 µl diluted monoclonal antibody (CD44-PE/Cy7 and CD24-PE, eBioscience) according to the manufacturer’s instructions. For detecting surface expression of SSEA-1, cells were counted and incubated with monoclonal antibody (CD15, Biolegend) following the manufacturer’s recommendations. After the incubation, cells were washed with PBS and analyzed through ACEA NovoCyteTM. FACS analysis was performed using Beckman moflo Astrios EQ. Cells with high (mcherry^high^) and low (mcherry^low^) B3GNT5 expression were sorted and seeded into ultralow-attachment 96-well plates with indicated cell number per well, respectively.

### Glycosylation analysis of B3GNT5 in vitro

To determine glycosylation patterns of B3GNT5 protein, cell lysates were treated with recombinant PNGase F (20411ES01, Yeasen Biotechnology) or O-glycosidase (P0733, New England BioLabs) following the manufacturer’s protocols. Briefly, B3GNT5-WT or B3GNT5-4NQ expressing cell lysates and glycoprotein denaturing buffer (1 M DTT and 5%SDS) were mixed gently, and proteins were denatured at 100 °C for 10 min. The denatured glycoproteins were mixed with indicated recombinant glycosidase as well as associated buffer, and then were deglycosylated at 37 °C for 1 ~ 2 h. After deglycosylation, B3GNT5 protein pattern was measured by western blotting.

### Methylation-specific PCR

To evaluate the methylation status of CpG sites within the B3GNT5 promoter, genomic DNA was extracted from each breast cancer cell line. 1 µg extracted DNA was bisulfite converted using the EpiTect Bisulfite Kit (Qiagen) according to the manufacturer’s instructions. Methylation-specific PCR reaction was performed using 2×EpiArt HS Taq kit (Vazyme) with the following nested primers:

Forward methylated primer: 5’-TTTTTTTCGTTGAGTAGAAGTTCGT-3’, Reverse methylated primer: 5’-AAAATATTTCCCTACCGCTCG-3’.

Forward unmethylated primer: 5’-TTTTTTTTGTTGAGTAGAAGTTTGT-3’, Reverse unmethylated primer: 5’- AAAAAATATTTCCCTACCACTCATC-3’.

20 ng bisulfite-converted DNA was used as the template. The PCR steps were as follows: 95 ℃ for 5 min, 29 cycles of 95 ℃ for 30 s, 58 ℃ for 30 s and 72 ℃ for 30 s, followed by a final extension at 72 °C for 5 min. The PCR product was analyzed by gel electrophoresis, and visualized images were captured.

### Western blot analysis

Cell lysates was prepared using RIPA buffer containing protease inhibitors (Roche), and protein concentration was measured by using Bradford assay kit (Fude Biological Technology). Adjusted protein samples were mixed with loading buffer and electrophoresed on 10% sodium dodecyl sulfate-polyacrylamide (SDS-PAGE) gels. After electrophoresis, proteins were transferred to polyvinylidene fluoride (PVDF) membranes. The membranes were blocked with 5% fat-free milk for 1 h at room temperature. Blocked membranes were incubated with indicated diluted primary antibody following manufacturer’s recommendations and gently shaking at 4 °C overnight. The blots were visualized using ECL assay after incubation with secondary antibody on the next day.

### Tumorigenesis assay and limiting dilution assay

Animal experiments were performed according to the approved procedures by the Institutional Animal Care and Use Committee at Zhejiang University. To determine the effect of B3GNT5 on *in vivo* tumorigenesis, female nude mice (5 ~ 6 weeks old) were injected with 1 × 10^6^ exogenous B3GNT5 knockout cells in the left flank and wild-type control cells in the right flank. Tumor formation was monitored every 2 ~ 4 d for 4 weeks. Tumor size and weight were measured. *In vivo* limiting dilution assay was performed using MDA-MB231 cells. Wild-type control cells and B3GNT5 knockout cells were diluted into different concentrations (1 × 10^5^, 1 × 10^4^ and 1 × 10^3^ cells per 100 µL) and injected into nude mice as previous. The frequency of stem cells was assessed by Extreme Limiting Dilution Analysis Program (http://bioinf.wehi.edu.au/software/elda/index.html.). Data were analyzed using the Student’s t-test; a *p*-value < 0.05 was considered significant.

### Statistical analysis

Results were expressed as mean ± SD or SEM as indicated. Comparisons were made by one-way ANOVA or the two-tailed Student’s t-test. Correlations were determined by Pearson’s correlation and Spearman’s rank correlation test. Survival curves were plotted using the Kaplan-Meier method, and differences were measured by the log-rank test. In all statistical tests, *p* < 0.05 was considered statistically significant.

## Results

### B3GNT5 is overexpressed in BLBC subtype

**Fig. 1:**
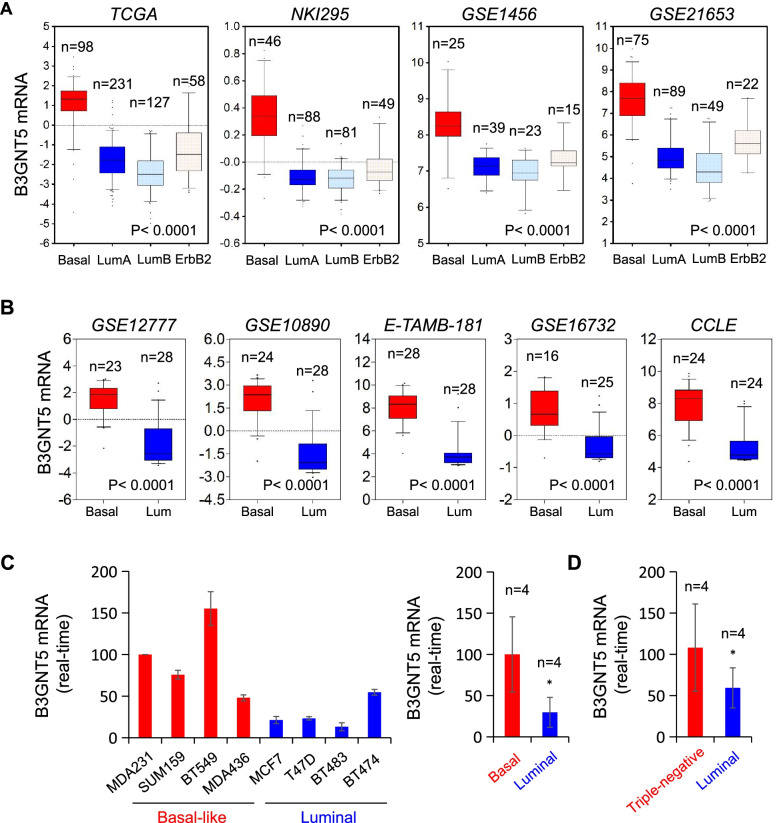
Elevated B3GNT5 expression was tightly associated with BLBC. (**A**) Box-plots indicated B3GNT5 mRNA expression in four different subtypes of breast cancer from four datasets (TCGA, NKI295, GSE1456 and GSE21653). (**B**) Box-plots indicated B3GNT5 mRNA expression in BLBC and luminal cell lines from different datasets (GSE12777, GSE16732, GSE10890, E-TAMB-181 and CCLE). Comparisons are made using the two-tailed Student’s t-test. (**C**) Expression of B3GNT5 mRNA was analyzed by quantitative real-time PCR in breast cancer cell lines (left panel), and the right panel showed the comparison analysis between basal-like and luminal breast cancer cell lines from left panel. Data are shown as mean ± SD based on three independent experiments. (**D**) Expression of B3GNT5 mRNA was analyzed by quantitative real-time PCR in clinical breast cancer samples. Data are shown as mean ± SD based on three independent experiments

Recently, we have reported several enzymes, such as fructose-1,6-biphosphatase (FBP1), urine diphosphate-galactose ceramide galactosyltransferase (UGT8) and phospholipid scramblase 1 (PLSCR1), were tightly correlated with breast cancer aggressiveness [20–22]. In order to investigate other possible molecules involving BLBC progression, we analyzed gene expression profiles in several publicly available gene expression datasets, including NKI295, TCGA, GSE1456, GSE21653, GSE22358 and METABRIC, which contain over 2500 breast cancer patients [23–25]. Apart from several known genes that were previously verified to have critical roles in BLBC, such as AKR1B1 [26] and UGT8. B3GNT5 mRNA expression was dramatically elevated in BLBC compared with other subtypes (Fig. 1 A and Figure S1A). To further characterize the correlation between B3GNT5 expression and basal subtype, we analyzed B3GNT5 mRNA expression in other five gene expression datasets, CCLE, E-TAMB-181, GSE10890, GSE16732, GSE12777, containing 48, 56, 52, 41 and 51 breast cancer cell lines, respectively [27–30]. Strikingly, high expression of B3GNT5 was associated with basal subtype of breast cancer cell lines (Fig. 1B and Figure S1B). In addition, we confirmed these findings by qRT-PCR in different subtypes of breast cancer cell lines and clinical samples, showing that B3GNT5 mRNA expression was significantly higher in basal subtype of breast cancer cell lines and triple-negative breast cancer samples that are mostly also BLBC (Fig. 1 C and D). These findings suggest that B3GNT5 overexpression is positively correlated with BLBC.

### B3GNT5 copy number amplification and hypomethylation of B3GNT5 promoter contribute to the overexpression of B3GNT5 in BLBC

**Fig. 2:**
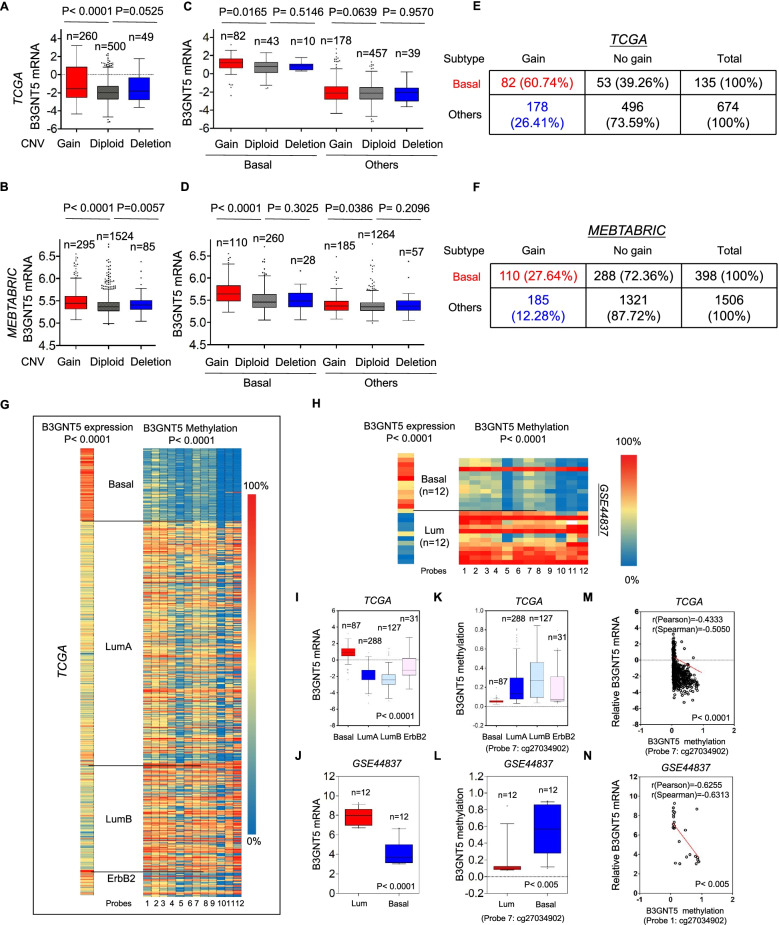
B3GNT5 overexpression correlated with its copy number amplification and promoter hypomethylation. **A**, **B** Box-plots indicated the correlation of B3GNT5 mRNA expression with its copy number variants status (gain, diploid and deletion) in breast cancer from the TCGA dataset (**A**) and MEBTABRIC dataset (**B**). **C**, **D** Box-plots showed the association of B3GNT5 mRNA level with copy number variants (gain, diploid and deletion) in different subtypes of breast cancer from the TCGA dataset (**C**) and MEBTABRIC dataset (**D**). **E**, **F** Analysis of the correlation between B3GNT5 mRNA expression and its copy number status (gain or no gain) in breast cancer from the TCGA dataset (**E**) and MEBTABRIC dataset (**F**). **G**, **H** Heat map displayed that B3GNT5 mRNA expression negatively correlated with promoter methylation of B3GNT5 using multiple 450 K probes in different subtypes of breast cancer (TGCA dataset) (**G**) and breast cancer cell lines (GSE44837 dataset) (**H**). **I**, **J** Box-plots indicated B3GNT5 mRNA expression in different subtypes of breast cancer (TGCA dataset) (**I**) and breast cancer cell lines (GSE44837 dataset) (**J**). **K**, **L** Box-plots indicated B3GNT5 promoter methylation in different subtypes of breast cancer (TGCA dataset) (**K**) and breast cancer cell lines (GSE44837 dataset) (**L**). **M**, **N** Analysis of the mRNA expression and methylation of B3GNT5 from the TCGA dataset (**M**) and GSE44837 dataset (**N**). The relative level of B3GNT5 mRNA was plotted against that of B3GNT5 methylation. Comparisons are made using the two-tailed Student’s t-test

Copy Number Variants (CNVs) are critical components of genetic variations composed of duplication, deletion, translocations and other chromosomal changes which strongly predispose to human cancer [31]. To determine the effect of CNVs on B3GNT5 expression, we analyzed the copy number alterations of breast cancer in TCGA, METABRIC and CCLE datasets. We found that cases with B3GNT5 amplification had significantly higher B3GNT5 expression than those with no amplification, supporting the notion that high B3GNT5 level might correlate with B3GNT5 copy number amplification (Fig. 2 A-B and Figure S2A). Additionally, we analyzed the copy number alterations in different subtypes of breast cancer tissues, observing that B3GNT5 copy number amplification was predominantly associated with BLBC subtype (Fig. 2 C-F and Figure S2B-C). These data strongly indicate that copy number amplification of B3GNT5 is closely associated with B3GNT5 overexpression and BLBC subtype.

Since many tumors with high expression of B3GNT5 did not have copy number amplification of B3GNT5, we assumed that other factors might link to this event. Aberrant DNA methylation, as an important regulator of gene transcription, controls gene expression, being an epigenetic hallmark of many types of cancer [32]. To determine whether B3GNT5 expression was affected by DNA methylation in breast tumors, we then analyzed methylation and expression of B3GNT5 from two datasets, TCGA and GSE44837. The correlation between B3GNT5 expression measured by gene expression microarray and B3GNT5 methylation assessed by 450 K Infinium microarray was analyzed. As expected, the promoter regions of B3GNT5 in BLBC had a remarkable reduction in methylation compared with other subtypes (Fig. 2G-H and Figure S2D-E). Notably, B3GNT5 mRNA expression was negatively correlated with B3GNT5 promoter methylation (Fig. 2I-N and Figure S2F-G). Additionally, Methylation-Specific PCR (MSP) analysis showed that the promoter regions of B3GNT5 in BLBC cell lines had a significant reduction in methylation compared with that in luminal breast cancer cell lines (Figure S2H). These data suggest that hypomethylation of B3GNT5 promoter is important for B3GNT5 overexpression.

### B3GNT5 tunes surface expression of SSEA-1

**Fig. 3:**
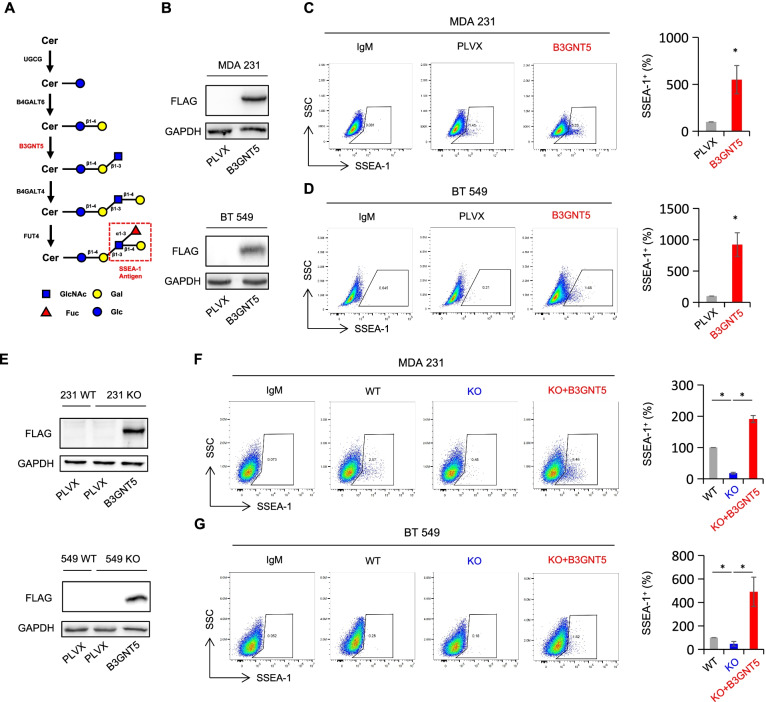
Surface SSEA-1 level was positively correlated with B3GNT5 expression. **A** The schematic diagram of SSEA-1 antigen formation. After the initiation of GSLs biosynthesis from ceramide (Cer) to glucosylceramide, B3GNT5 is responsible for Lc3Cer formation. Elongation of Lc3Cer by β-1,4-galactosyltransferase 4 (B4GALT4) and α-1,3-fucosyltransferase 4 (FUT4) yields the SSEA-1 antigen. **B** Expression of B3GNT5 was examined by Western blotting in MDA-MB231 and BT549 cells with stable empty vector or B3GNT5-WT-Flag cDNA. **C**, **D** Surface expression of SSEA-1 antigen was tested by flow cytometry in MDA-MB231 (**C**) and BT549 cells (**D**) with stable empty vector or B3GNT5-WT-Flag cDNA. **E** Expression efficiency of B3GNT5 was analyzed by Western blotting in wild-type MDA-MB231 and BT549 cells (WT) as well as B3GNT5-knockout MDA-MB231 and BT549 cells with stable empty vector (KO) and B3GNT5-WT-Flag cDNA (KO + B3GNT5). **F**, **G** Flow cytometry measuring surface SSEA-1 level in the indicated cell lines. Data are presented as the percentage of wild-type vector cell line. *p < 0.05 by Student’s t test. Data are shown as mean ± SD based on three independent experiments

B3GNT5 is the sole enzyme catalyzing lactosylceramide into Lc3Cer, and plays a pivotal role in synthesis of (neo-) lacto-series glycosphingolipids (GSLs). Thus, we sought to determine whether B3GNT5 could metabolically regulate SSEA-1 expression, a lacto-series carbohydrate structure which overexpressed in triple-negative breast cancer (Fig. 3 A) [33]. We established stable transfectants with empty vector or B3GNT5-WT-Flag expression plasmid in MDA-MB231 and BT549 cells (Fig. 3B). Flow cytometry analysis showed that expression of B3GNT5 significantly elevated the SSEA-1 level (Fig. 3 C-D). To gain further insight into the involvement of B3GNT5 expression in regulating surface SSEA-1 level, we utilized genome editing strategy, CRISPR-Cas9, to knockout B3GNT5 in MDA-MB231, BT549 and SUM159 cell lines. Edited sequence at the Cas9-active sites were detected by DNA sequencing (Figure S3A). As expected, surface expression of SSEA-1 in MDA-MB231 KO and BT549 KO cells was dramatically down-regulated compared with that in wild-type control cells. However, re-expressing B3GNT5 in these KO cell lines remarkably rescued the expression of SSEA-1 (Fig. 3E-G). Together, these data indicate that B3GNT5 contributes to the surface expression of SSEA-1.

### B3GNT5 contributes to the maintenance of CSCs

**Fig. 4:**
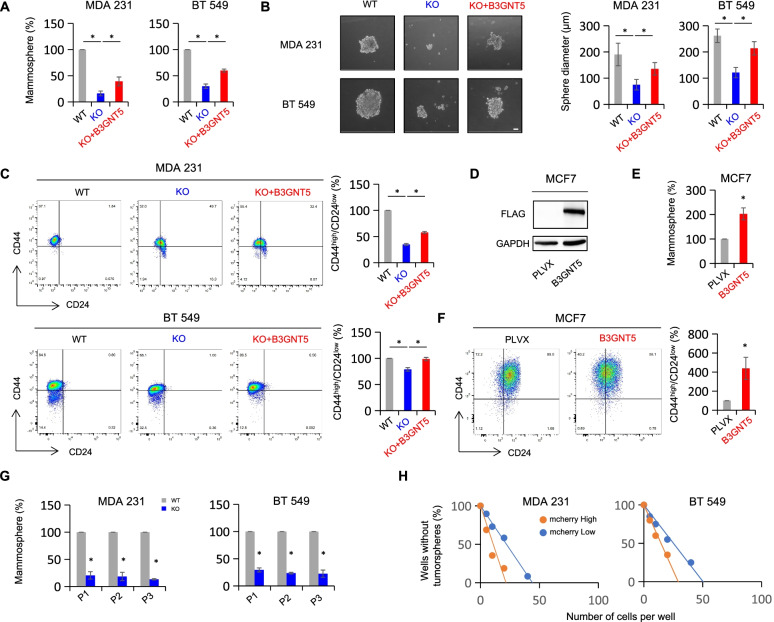
B3GNT5 promoted mammosphere formation and enhances CSC population. **A**, **B** Mammosphere formation of BT549 and MDA-MB231 cells with or without B3GNT5 knockout as well as stably B3GNT5-expressing KO cells (**A**), and representative images are shown (**B**). Scale bars, 50 μm. (**C**) Populations of CSCs (CD44^high^/CD24^low^) were measured by flow cytometry in MDA-MB231 and BT549 cells with or without B3GNT5 knockout as well as stably B3GNT5-expressing KO cells. **D** Expression of B3GNT5 was analyzed by Western blotting in MCF7 cells with stable empty vector or B3GNT5 expression. **E**, **F** Mammosphere formation (**E**) and population of CSCs (CD44^high^/CD24^low^) (**F**) were examined in MCF7 cells with stable empty vector or B3GNT5-WT-Flag cDNA. **G** Sequential mammosphere formation were measured in MDA-MB231 and BT549 cells with or without B3GNT5 knockout as well as stably B3GNT5-expressing KO cells. P1, P2 and P3 indicated primary, secondary and tertiary passage of mammosphere, respectively. **H** Limiting dilution sphere forming assay was performed by fluorescence-activated cell sorting in MDA-MB231 KO and BT549 KO cells with B3GNT5-WT-mcherry overexpression. Cells with high (mcherry^high^) and low (mcherry^low^) B3GNT5 expression were sorted separately. Data are presented as the percentage of wild-type or vector cell lines. *p < 0.05 by Student’s t test. Data are shown as mean ± SD based on three independent experiments

In addition to the role of SSEA-1 as a tumor-initiating marker in glioblastoma multiforme cells [12], another recent study showed that B3GNT5 was correlated with glioma stem cells, and its expression was significantly downregulated during glioma stem cells differentiation [8]. Given the importance of B3GNT5 and SSEA-1 in cancer cell stemness, B3GNT5 might be required for the maintenance of breast cancer cell stemness. To verify this notion, we first examined the impact of B3GNT5 expression on proliferation in MDA-MB231, BT549 and SUM159 cells. Knockout of B3GNT5 significantly suppressed the proliferation in these cells, whereas ectopic expression of B3GNT5 remarkably restored the decreased proliferation rate (Figure S4A). Next, we assessed the effects of B3GNT5 knockout on the mammosphere formation. Strikingly, B3GNT5 knockout dramatically impeded the mammosphere formation in MDA-MB231, BT549 and SUM159 cells, which was partially rescued in MDA-MB231 KO and BT549 KO cells by ectopic expression of B3GNT5 (Fig. 4 A-B and Figure S4B), whereas ectopic B3GNT5 expression in MCF7 cells led to a remarkable promoted mammosphere formation (Fig. 4D-E). Human breast CSCs are enriched in cells of CD44^high^/CD24^low^ population [34]. To explore correlation between B3GNT5 expression and breast CSCs, we analyzed the potential changes of CD44^high^/CD24^low^ cells populations by flow cytometry. Similar to the observation in mammosphere formation, B3GNT5 knockout led to a significant reduction of CD44^high^/CD24^low^ populations in MDA-MB231, BT549 and SUM159 cells, whereas ectopic expression of B3GNT5 partially rescued the CD44^high^/CD24^low^ populations in MDA-MB231 KO and BT549 KO cells (Fig. 4 C and Figure S4C). Consistently, ectopic B3GNT5 expression also increased CD44^high^/CD24^low^ population in MCF7 cells (Fig. 4 F). Subsequent resuspending and replating of cells from the primary and secondary mammospheres showed a similar reduced spheres formation in MDA-MB231 KO and BT549 KO cells compared with wild-type control cells (Fig. 4G). Using *in vitro* limiting dilution assay, we observed that MDA-MB231 KO and BT549 KO cells with higher expression of B3GNT5 were consistently more tumorigenic (Fig. 4 H, Figure S4D and Figure S4E). These results suggest that B3GNT5 expression is important for stemness maintenance of breast cancer cells.

### B3GNT5 is required for tumorigenicity of breast cancer

**Fig. 5:**
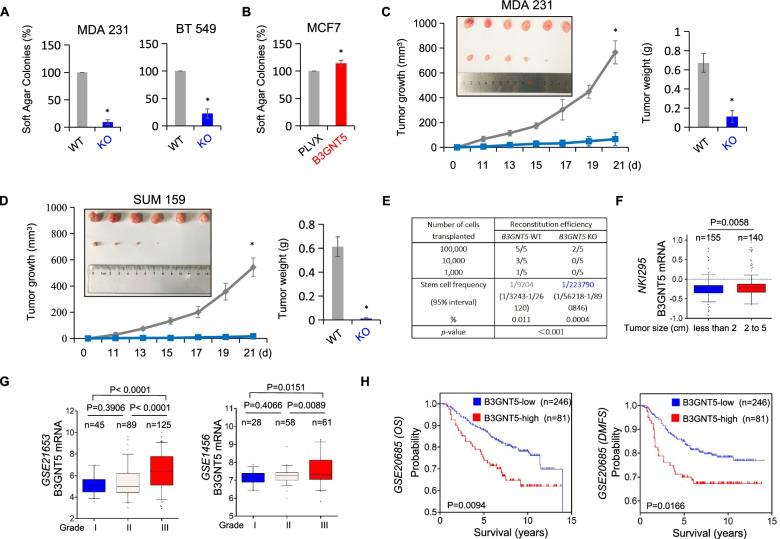
Knockout of B3GNT5 suppressed tumorigenicity ***in vitro*** and ***in vivo***, and elevated B3GNT5 expression predicts poor clinical outcomes. **A**, **B** Soft-agar assay was performed using MDA-MB231 and BT549 cells with or without B3GNT5 knockout (**A**) as well as MCF7 cells with stable empty vector or B3GNT5-WT-Flag cDNA (**B**). Data are presented as the percentage of wild-type or vector cell lines. Data are shown as mean ± SD based on three independent experiments. **C**, **D** MDA-MB231 (**C**) and SUM159 cells (**D**) with or without B3GNT5 knockout were injected into the mammary fat pad of nude mice. Tumor growth was measured every two days. On day 21, mice were sacrificed and tumor weights were recorded. Data are presented as mean ± SEM of six mice. *p < 0.05 by Student’s t test. **E** MDA-MB231 cells with or without B3GNT5 knockout were transplanted into the mammary fat pad of nude mice. 12 weeks after transplantation, tumor formation was analyzed by extreme limiting dilution analysis. **F** Box-plots indicated B3GNT5 expression in different tumor sizes of breast cancer from NKI295 dataset. Comparisons are made using the two-tailed Student’s t-test. **G** Box-plots indicated B3GNT5 expression in different histological grades of breast cancer from GSE21653 and GSE1456 datasets. Comparisons between two groups are made using the two-tailed Student’s t-test. **H** Kaplan-Meier survival analysis for OS and DMFS of patients in GSE20685 dataset according to B3GNT5 expression status. The p-value is determined using the log-rank test

Having identified the association of B3GNT5 expression with CSCs, we then evaluated the effect of B3GNT5 expression on the *in vitro* tumorigenicity using soft agar assay. B3GNT5 knockout led to a dramatic reduction of colonies in MDA-MB231, BT549 and SUM159 cells, whereas B3GNT5 expression enhanced the ability of colony formation in MCF-7 cells (Fig. 5 A-B and Figure S5A). Subsequently, we examined tumorigenicity using xenograft models. Markedly, MDA-MB231 and SUM159 cells with B3GNT5 knockout significantly reduced tumor growth *in vivo* compared with wild-type control cells (Fig. 5 C-D). Similarly, *in vivo* limiting dilution assay also revealed that MDA-MB231 cells with B3GNT5 knockout had a relatively low incidence of tumorigenesis (Fig. 5E). To further explore the clinical relevance of B3GNT5 expression in breast cancer progression, we first assessed the association of B3GNT5 expression with tumor size in NKI295 dataset in which 295 patients were separated into two groups based on the primary tumor size. Remarkably, high B3GNT5 expression was correlated with a larger tumor size (Fig. 5 F). Furthermore, we determined the correlation between B3GNT5 expression and histological grades of breast cancer patients. By using five datasets (GES21653, GES22358, GES1456, NKI295 and MEBTABRIC), in which patients were scored for the tumor grades, we found that B3GNT5 was predominantly expressed in high tumor grade, especially in third grade (Fig. 5G and Figure S5B). Finally, we sought to elucidate the association between B3GNT5 expression and patient survival in GSE20685 dataset and an aggregate breast cancer dataset by Kaplan-Meier survival analysis [23, 24, 35]. The analysis showed that patients with high expression of B3GNT5 had shorter overall survival (OS), relapse-free survival (RFS) and distant metastasis-free survival (DMFS) (Fig. 5 H and Figure S5C). These clinical data support the critical role of B3GNT5 in breast cancer aggressiveness.

### Glycosylation of B3GNT5 is important for its protein stabilization

**Fig. 6:**
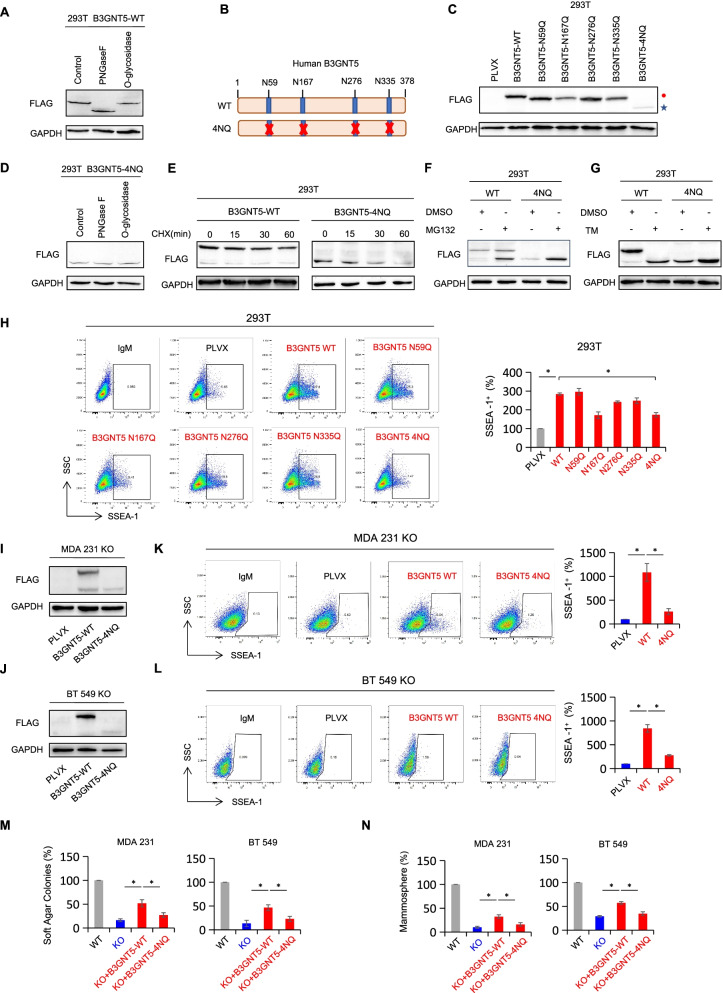
B3GNT5 was N-glycosylated, and glycosylation of B3GNT5 stabilized its protein. **A** Cell lysates from HEK293T cells expressing B3GNT5-WT-Flag were treated with PNGase F or O-glycosidase for 2 h at 37 °C *in vitro*. Band shift was detected by Western blotting. **B** Schematic diagram of each asparagine mutated site and human B3GNT5-4NQ mutant used in this study. **C** Expression pattern of each asparagine mutated site was examined by Western blotting in HEK293T cells. Red closed circle, glycosylated B3GNT5 (B3GNT5-WT); blue star, non-glycosylated B3GNT5 (B3GNT5-4NQ). **D** Cell lysates from HEK293T with B3GNT5-4NQ expression was treated with PNGase F or O-glycosidase for 2 h at 37 °C *in vitro*. Band shift was detected by Western blotting. **E** HEK293T cells with B3GNT5-WT or B3GNT5-4NQ expression were treated with CHX (20 µM) for indicated intervals. **F** HEK293T cells with B3GNT5-WT or B3GNT5-4NQ expression were treated with MG132 (20 µM) for 8 h. **G** HEK293T cells with B3GNT5-WT or B3GNT5-4NQ expression were treated with tunicamycin (TM, 2.5 µg/mL) for 24 h. B3GNT5 protein level was detected using Western blotting. **H** HEK293T cells with stable empty vector, wild-type B3GNT5 or different mutants were used for measuring surface level of SSEA-1. Data are shown as a percentage of control cell line. Data are shown as mean ± SD based on three independent experiments. **I**, **J** Expression efficiency of B3GNT5-WT-Flag and B3GNT5-4NQ-Flag was measured in MDA-MB231 KO (**I**) and BT549 KO cells (**J**). **K**, **L** Surface expression of SSEA-1 was detected using flow cytometry in indicated cell lines. **M**, **N** Soft-agar assay (**M**) and mammosphere assay (**N**) were performed using empty vector, B3GNT5-WT-Flag or B3GNT5-4NQ-Flag re-expressing MDA-MB231 KO cells and BT549 KO cells. Data are presented as a percentage of control cell lines as in (**H**)

When analyzing exogenous B3GNT5 protein expression efficiency in cells with B3GNT5-WT-Flag expression, we surprisingly noticed the immunoblotting band of B3GNT5 protein at ~ 52 kDa (the predicted molecular weight is around 44 kDa) (Fig. 3B). According to the previous findings, glycosylated proteins usually show heterogeneous patterns and display higher molecular weights than predicted ones through immunoblots [36]. To determine whether B3GNT5 is a glycosylated protein and to verify its potential glycosylation pattern, we treated HEK293T cell lysates with recombinant peptide-N-glycosidase F (PNGase F), an N-glycan cleaving glycosidase, and recombinant O-glycosidase that removes core 1 and core 3 O-linked disaccharides from glycoproteins *in vitro*. Intriguingly, cell lysates treated with PNGase F showed an obvious band shift compared with control group (from ~ 52 kDa to ~ 44 kDa). However, the addition of O-glycosidase did not display the similar result. These findings suggest that B3GNT5 might be predominantly modified as N-linked glycosylated protein (Fig. 6 A). To pinpoint the glycosylation sites, we searched for NXT motifs in human-derived amino-acid sequence of B3GNT5, and noticed four sites (Fig. 6B). To further validate B3GNT5 protein glycosylation, we generated B3GNT5-WT-Flag expressing plasmid with sites mutation and tested expression efficiency by Western blotting. Compared with B3GNT5-WT-Flag expressing group, a slight and similar reduction of molecular weight was observed when single asparagine (N) was substituted to glutamine (Q) (N59Q, N167Q, N276Q and N335Q) (Fig. 6 C), indicating that each of the N-glycosylated site was modified. When all four asparagines were mutated into glutamines (4NQ), the band detected by Western blotting reduced to ~ 44 kDa (Fig. 6 C), which was approximate to the theoretical molecular weight of B3GNT5 protein. Additionally, N-linked glycosylation inhibitor tunicamycin (TM) treatment almost completely inhibited glycosylation of B3GNT5, showing the same band position with B3GNT5-4NQ (Fig. 6G). Following treating lysates of HEK293T expressing B3GNT5-4NQ-Flag with PNGase F or O-glycosidase, no apparent mobility shift was observed (Fig. 6D). These data suggest that N-linked glycan other than O-linked glycan structure predominantly exists in B3GNT5 protein and 4 asparagine sites (N59, N167, N276 and N335) are responsible for glycosylation of B3GNT5.

Since the expression of glycosylated B3GNT5 was significantly higher than that of non-glycosylated form (Fig. 6 C), we then sought to verify whether glycosylation affect protein stability of B3GNT5. Following treating with protein synthesis inhibitor cycloheximide (CHX), non-glycosylated B3GNT5 protein degraded more rapidly than glycosylated form in HEK293T cells (Fig. 6E). To determine whether proteasome might involve in this process, we then treated glycosylated and non-glycosylated B3GNT5 with proteasome inhibitor MG132. We observed that non-glycosylated B3GNT5 protein was significantly rescued in the presence of MG132, whereas glycosylated B3GNT5 protein was rescued marginally (Fig. 6 F). Next, we evaluated the effect of B3GNT5 protein N-glycosylation on surface SSEA-1 level in HEK293T cells with mutated N-glycosylation sites. As expected, ectopic wild-type B3GNT5 dramatically increased, whereas B3GNT5-4NQ impeded the surface SSEA-1 expression (Fig. 6 H), suggesting that surface SSEA-1 expression was positively correlated with glycosylation and protein level of B3GNT5. It is noteworthy that TM treatment resulted in a remarkable decrease of B3GNT5 protein level in B3GNT5-expressing MDA-MB231 and BT549 cells (Figure S6A), whereas MG132 caused a significant increase in non-glycosylated B3GNT5 protein level (Figure S6B). Additionally, B3GNT5-4NQ expression in MDA-MB231 KO and BT549 KO cells significantly increased surface SSEA-1 expression; however, this increment was much lower than in B3GNT5-WT expressing KO cells (Fig. 6I-L). Consistently, enhancement of sphere-forming ability by B3GNT5-4NQ expression was much weaker than that by B3GNT5-WT expression in MDA-MB231 KO and BT549 KO cells (Fig. 6 N-M). Together, these results suggest that N-glycosylation of B3GNT5 is critical for its protein stability, contributing to pro-tumorigenic activity of B3GNT5.

## Discussion

In this study, we report that B3GNT5 contributes to the stem cell properties and tumorigenic ability of BLBC, providing new insights into the critical role of B3GNT5 in BLBC.

### B3GNT5 copy number amplification and hypomethylation of B3GNT5 promoter contribute to the overexpression of B3GNT5 in BLBC

CNVs have been studied as important determinants for many types of cancer. Several CNVs that are correlated with poor outcomes have been studied in BLBC [31, 37, 38]. Our recent study has demonstrated that HIST1H1B overexpression was partially due to the copy number amplification. CNV is usually considered as a critical factor that involves in the changes of mRNA expression. Indeed, we identified that cases with copy number amplification of B3GNT5 had a significantly higher B3GNT5 expression than those with no amplification by analyzing copy number variation in different subtypes of breast cancer tissues and cell lines from TCGA, METABRIC and CCLE datasets. Additionally, cases with B3GNT5 copy number amplification were positively correlated with BLBC subtype. These results indicate that B3GNT5 copy number amplification is responsible for high B3GNT5 expression, especially in BLBC.

Some breast tumors with B3GNT5 overexpression were observed to have no B3GNT5’s copy number amplification, indicating the involvement of other genetic or epigenetic mediators in the upregulation of B3GNT5 expression. DNA hypermethylation causes gene silencing [39], whereas DNA hypomethylation is tightly associated with transcription activation [40]. DNA hypomethylation can mediate downstream tumor-associated signaling pathway by transcription activation of oncogenes [41, 42]. Our data demonstrated that low methylation level in promoter regions of B3GNT5 in breast cancer, especially in BLBC, had a positive correlation with high expression of B3GNT5 through analyzing B3GNT5 methylation datasets and gene expression microarray. Together, these findings suggest that the hypomethylation of B3GNT5 promoter is another critical mediator for the upregulation of B3GNT5 expression.

### B3GNT5 contributes to surface expression of SSEA-1 and CSCs properties in BLBC

Cellular glycosylation pattern changes have been verified as important steps in embryonic development [4, 43–45]. These carbohydrate structures, as well as some glycan-binding proteins, also precisely control many key processes of tumor progression [45–47]. SSEA-1, as one of the Lewis blood antigens, has been shown to participate in embryonic development and cancer progression [12, 48]. A recent study has revealed that expression of SSEA-1 is associated with poor prognosis in TNBC, especially in younger patients [33]. More studies mostly focused on the effect of SSEA-1, as a potential stem cell marker in various cancers [10, 12, 17]. Our data showed that B3GNT5, as the key enzyme responsible for (neo-) lacto-series GSLs synthesis, elevated surface expression of SSEA-1 in breast cancer cells.

Accumulating evidence has verified BLBC has enhanced CSCs properties compared with other breast cancer subtypes [34, 49, 50]. Interestingly, B3GNT5 expression is essential for embryonic development [4], implying the possible involvement of B3GNT5 in cell stemness. Given the tight association among B3GNT5, SSEA-1 and CSCs, we speculated that B3GNT5 might regulate breast CSCs properties. Indeed, our data showed that B3GNT5 expression in luminal cells enhanced, whereas B3GNT5 knockout in BLBC cells suppressed CSCs properties *in vitro*. Cancer stem cells contribute to tumor growth and development [51–53]. Consistently, our data showed that B3GNT5 expression promoted tumorigenesis. Clinically, BLBC patients had high expression of B3GNT5, and elevated B3GNT5 expression predicted poor overall survival. These results provide a positive correlation between B3GNT5 expression and aggressiveness of BLBC cells.

### Glycosylation of B3GNT5 is critical for its protein stabilization

Aberrant glycosylation in glycoproteins has been regarded to have key roles in tumor biology. Increasing studies have revealed that protein stabilization is closely regulated by its protein glycosylation, such as PD-L1 glycoprotein [36, 54]. In this study, we found that glycosylation of B3GNT5 stabilized its protein level by inhibiting 26 S-mediated protein degradation. We further identified N59, N167, N276 and N335 as the four unique N-glycosylation sites of B3GNT5, as observed that these mutations in four NXT motifs dramatically reduced B3GNT5 protein molecular weight and stability. Additionally, we revealed that non-glycosylated form of B3GNT5 protein caused significantly less surface SSEA-1 expression and CSCs properties than its glycosylated form. Together, these results support the critical role of B3GNT5 glycosylation in its protein stability and pro-tumorigenic effect.

### B3GNT5 represents a potential prognostic indicator and therapeutic target for BLBC

Since B3GNT5 expression was tightly associated with breast cancer, it was critical to assess whether B3GNT5 is appropriate for breast cancer patients’ diagnosis. We have identified several factors that might predict patient prognosis, including (1) Breast cancer subtypes: B3GNT5 expression is especially elevated in BLBC; (2) Tumor size: high expression of B3GNT5 is correlated with larger tumor size; (3) Tumor grade: high expression of B3GNT5 is associated with high tumor grade; (4) Survival rate: high expression of B3GNT5 predicts poor survival in breast cancer patients. These findings strongly support that B3GNT5 is a promising prognostic biomarker for breast cancer patients.

## Conclusions

Our study showed that B3GNT5 enhanced CSCs properties in BLBC, which may be especially important due to ineffectiveness of traditional cancer therapies against the CSCs that regenerate tumors [55]. In addition, accumulating studies have been focusing on the aberrant glycosylation of glycoproteins as well as glycolipids in various types of cancer progression, suggesting that identifying the specific glycosyltransferases responsible for tumor aggressiveness is pressingly needed. Given a close association of B3GNT5 overexpression and glycosylation with increased CSCs properties and tumorigenicity in BLBC, implying that B3GNT5 and the corresponding glycosyltransferases may have the potential to become therapeutic targets of BLBC.

## Supplementary information

**Additional file 1: Figure S1.** A Box-plots indicated B3GNT5 mRNA expression in four different subtypes of breast cancer from the GSE22358 and MEBTABRIC datasets. Comparisons are made using the two-tailed Student's t-test. B Histograms indicated B3GNT5 mRNA expression in different luminal and BLBC cell lines from four datasets (GSE12777, GSE16732, GSE10890 and E-TAMB-181)

**Additional file 2: Figure S2.** A Box-plots indicated the correlation of B3GNT5 mRNA expression with its copy number variants status (gain, diploid and deletion) in breast cancer from the CCLE dataset. B Box-plots showed the association of B3GNT5 mRNA level with copy number variants (gain, diploid and deletion) in different subtypes of breast cancer from the CCLE dataset. C Analysis of the correlation between B3GNT5 mRNA expression and its copy number status (gain or no gain) in breast cancer from the CCLE dataset. (D, E) Box-plots indicated B3GNT5 promoter methylation in different subtypes of breast cancer (TCGA dataset) (D), and breast cancer cell lines (GSE44837 dataset) (E). (F, G) Analysis of the mRNA expression and methylation of B3GNT5 from the TCGA dataset (F), and GSE44837 dataset (G), The relative level of B3GNT5 mRNA was plotted against that of B3GNT5 methylation. Comparisons are made using the two-tailed Student's t-test. The relative level of B3GNT5 mRNA was plotted against that of B3GNT5 methylation. Comparisons are made using the two-tailed Student's t-test. H Analysis of the methylation status of B3GNT5 promoter regions in different subtypes of breast cancer cell lines

**Additional file 3: Figure S3.** A B3GNT5 knockout in MDA-MB231, BT549 and SUM159 cells using CRISPR–Cas9 technology. Knockout results were verified by DNA sequencing

**Additional file 4: Figure S4.** A Growth of indicated cell lines with stable empty vector or B3GNT5-WT-Flag re-expression was measured by cell-count assay for 2 days.B Mammosphere formation of SUM159 cells with or without B3GNT5 knockout. C Population of CSCs (CD44high/CD24low) was analyzed by flow cytometry in SUM159 cells with or without B3GNT5 knockout. Data are shown as a percentage of WT cell line. *p< 0.05 by Student’s t test. Data are shown as mean ± SD based on three independent experiments. D Expression of B3GNT5 was detected by Western blotting in MDA-MB231 KO and BT549 KO cells with stable empty vector or B3GNT5-WT-mcherry cDNA. E Extreme limiting dilution analysis of sphere formation in B3GNT5 expressing MDA-231 KO and BT549 KO cell lines in vitro

**Additional file 5: Figure S5.** A Soft-agar assay was performed using SUM159 cells with or without B3GNT5 knockout. Data are shown as a percentage of WT cell line. *p< 0.05 by Student’s t test. Data are shown as mean ± SD based on three independent experiments. B Box-plots indicated B3GNT5 mRNA expression in different histological grades of breast cancer from GSE22358, NKI295 and MEBTABRIC datasets. Comparisons between two groups are made using the two-tailed Student’s t-test. C Kaplan-Meier survival analysis for OS, RFS and DMFS of patients in an aggregate breast cancer dataset according to B3GNT5 expression status. The p-value is determined using the log-rank test

**Additional file 6: Figure S6.** A MDA-MB231 and BT549 cells with B3GNT5-WT-mcherry expression were treated with TM (2.5 μg/mL) for 24 h. B MDA-MB231 and BT549 cells expressing B3GNT5-WT-Flag or B3GNT5-4NQ-Flag were treated with MG132 (20 μM) for 8 h. B3GNT5 protein level was detected by Western blotting

## Data Availability

The microarray datasets that were utilized in the study were retrieved from the NIH-GEO dataset database (http://www.ncbi.nlm.nih.gov/gds/), EMBL-EBI dataset database (https://www.ebi.ac.uk/) and Cancer Cell Line Encyclopedia (https://sites.broadinstitute.org/ccle). Information about TCGA and the investigators and institutions that constitute the TCGA research network can be found at http://cancergenome.nih.gov/. Other data generated during this study are included in this published article.

## Abbreviations

B3GNT5: β1,3-N-acetylglucosaminyltransferase V
BLBC: Basal-like breast cancer
CSCs: Cancer stem cells
GSLs: Glycosphingolipids
SSEA-1: Stage-specific embryonic antigen 1
Lc3Cer: Lactotriosylceramide
CNVs: Copy number variants
RT-qPCR: Real-time quantitative polymerase chain reaction
MSP: Methylation-specific PCR
TM: Tunicamycin
CHX: Cycloheximide
DMFS: Distant metastasis-free survival
OS: Overall survival
RFS: Relapse-free survival
