# Supplementary material for: Elevated transcription and glycosylation of B3GNT5 promotes breast cancer aggressiveness
Source: J Exp Clin Cancer Res. 2022 May 7;41:169. doi: 10.1186/s13046-022-02375-5 (PMC9077843; doi:10.1186/s13046-022-02375-5)
Supplement: Supplementary file 1 — Additional file 1: Figure S1. A Box-plots indicated B3GNT5 mRNA expression in four different subtypes of breast cancer from the GSE22358 and MEBTABRIC datasets. Comparisons are made using the two-tailed Student's t-test. B Histograms indicated B3GNT5 mRNA expression in different luminal and BLBC cell lines from four datasets (GSE12777, GSE16732, GSE10890 and E-TAMB-181) [file 13046_2022_2375_MOESM1_ESM.pdf]

Supplementary Materials

Supplementary Figure Legends

Figure S1

(A) Box-plots indicated B3GNT5 mRNA expression in four different subtypes of breast cancer from the GSE22358 and MEBTABRIC datasets. Comparisons are made using the two-tailed Student's t-test.

(B) Histograms indicated B3GNT5 mRNA expression in different luminal and BLBC cell lines from four datasets (GSE12777, GSE16732, GSE10890 and E-TAMB-181).

Figure S2

(A) Box-plots indicated the correlation of B3GNT5 mRNA expression with its copy number variants status (gain, diploid and deletion) in breast cancer from the CCLE dataset.

(B) Box-plots showed the association of B3GNT5 mRNA level with copy number variants (gain, diploid and deletion) in different subtypes of breast cancer from the CCLE dataset.

(C) Analysis of the correlation between B3GNT5 mRNA expression and its copy number status (gain or no gain) in breast cancer from the CCLE dataset.

(D, E) Box-plots indicated B3GNT5 promoter methylation in different subtypes of breast cancer (TCGA dataset) (D) and breast cancer cell lines (GSE44837 dataset) (E).

(F, G) Analysis of the mRNA expression and methylation of B3GNT5 from the TCGA dataset (F) and GSE44837 dataset (G). The relative level of B3GNT5 mRNA was plotted against that of B3GNT5 methylation. Comparisons are made using the two-tailed Student's t-test. The relative level of B3GNT5 mRNA was plotted against that of B3GNT5 methylation. Comparisons are made using the two-tailed Student's t-test.

(H) Analysis of the methylation status of B3GNT5 promoter regions in different subtypes of breast cancer cell lines.

Figure S3

(A) B3GNT5 knockout in MDA-MB231, BT549 and SUM159 cells using CRISPR–Cas9 technology. Knockout results were verified by DNA sequencing.

Figure S4

(A) Growth of indicated cell lines with stable empty vector or B3GNT5-WT-Flag re-expression was measured by cell-count assay for 2 days.

(B) Mammosphere formation of SUM159 cells with or without B3GNT5 knockout.

(C) Population of CSCs ($CD44^{high}/CD24^{low}$) was analyzed by flow cytometry in SUM159 cells with or without B3GNT5 knockout. Data are shown as a percentage of WT cell line. *$p< 0.05$ by Student's t test. Data are shown as mean ± SD based on three independent experiments.

(D) Expression of B3GNT5 was detected by Western blotting in MDA-MB231 KO and BT549 KO cells with stable empty vector or B3GNT5-WT-mcherry cDNA.

(E) Extreme limiting dilution analysis of sphere formation in B3GNT5 expressing MDA-231 KO and BT549 KO cell lines *in vitro*.

Figure S5

(A) Soft-agar assay was performed using SUM159 cells with or without B3GNT5 knockout. Data are shown as a percentage of WT cell line. *$p< 0.05$ by Student's t test. Data are shown as mean ± SD based on three independent experiments.

(B) Box-plots indicated B3GNT5 mRNA expression in different histological grades of breast cancer from GSE22358, NKI295 and MEBTABRIC datasets. Comparisons between two groups are made using the two-tailed Student's t-test.

(C) Kaplan-Meier survival analysis for OS, RFS and DMFS of patients in an aggregate breast cancer dataset according to B3GNT5 expression status. The p-value is determined using the log-rank test.

Figure S6

(A) MDA-MB231 and BT549 cells with B3GNT5-WT-mcherry expression were treated with TM (2.5 μg/mL) for 24 h.

(B) MDA-MB231 and BT549 cells expressing B3GNT5-WT-Flag or B3GNT5-4NQ-Flag were treated with MG132 (20 μM) for 8 h. B3GNT5 protein level was detected by Western blotting.

Supplementary Figures

Figure S1

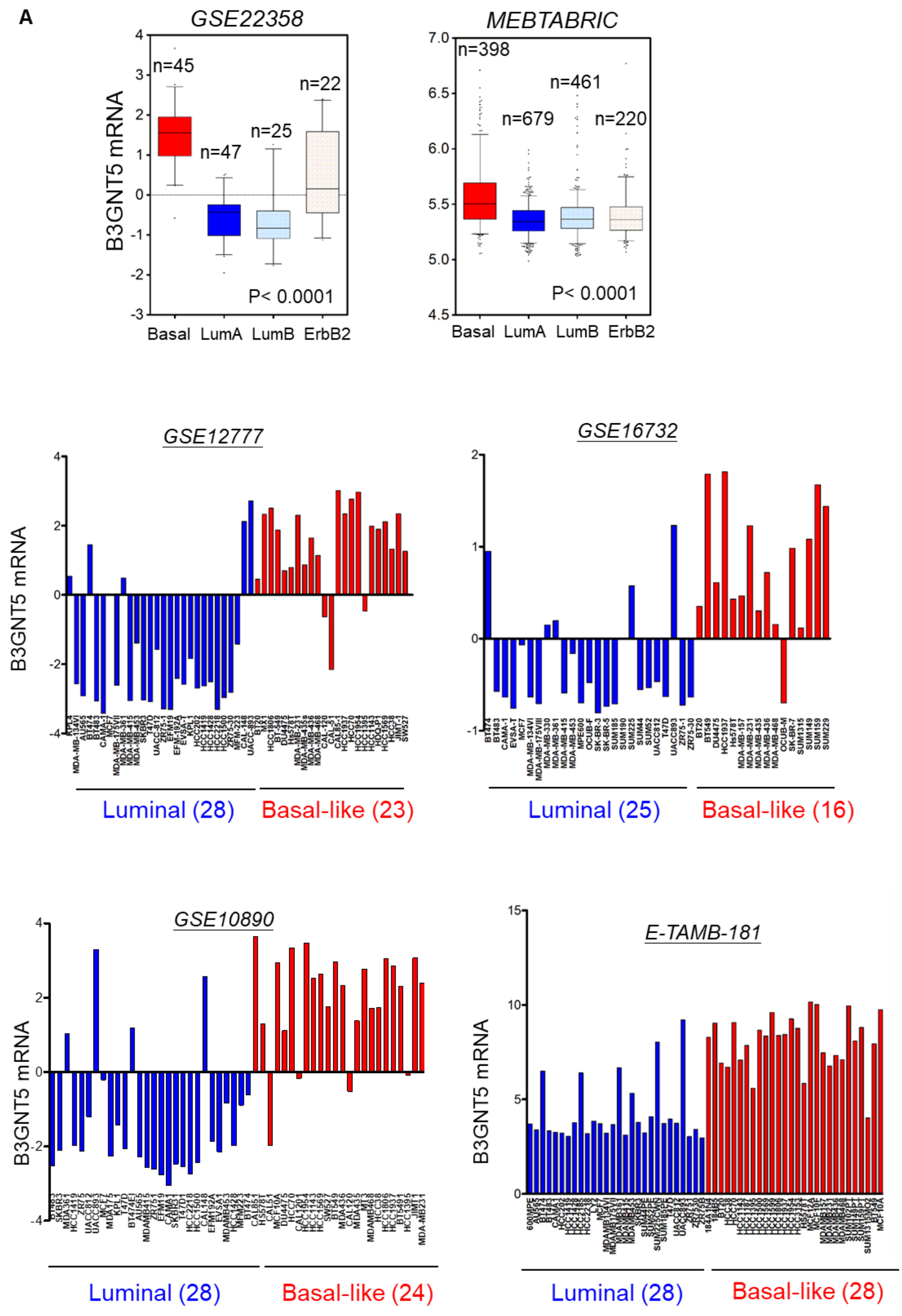

Figure S2

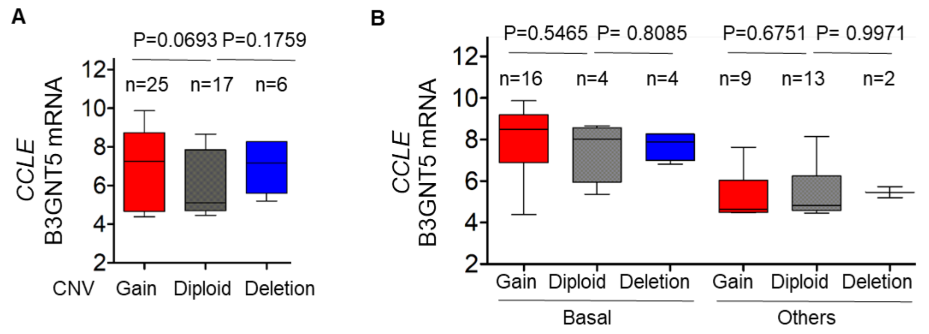

C

Subtype	Gain	*CCLE* (n=48)<br>No gain	Total
Basal	16 (66.67%)	8 (33.33%)	24 (100%)
Others	9 (37.5%)	15 (62.5%)	24 (100%)

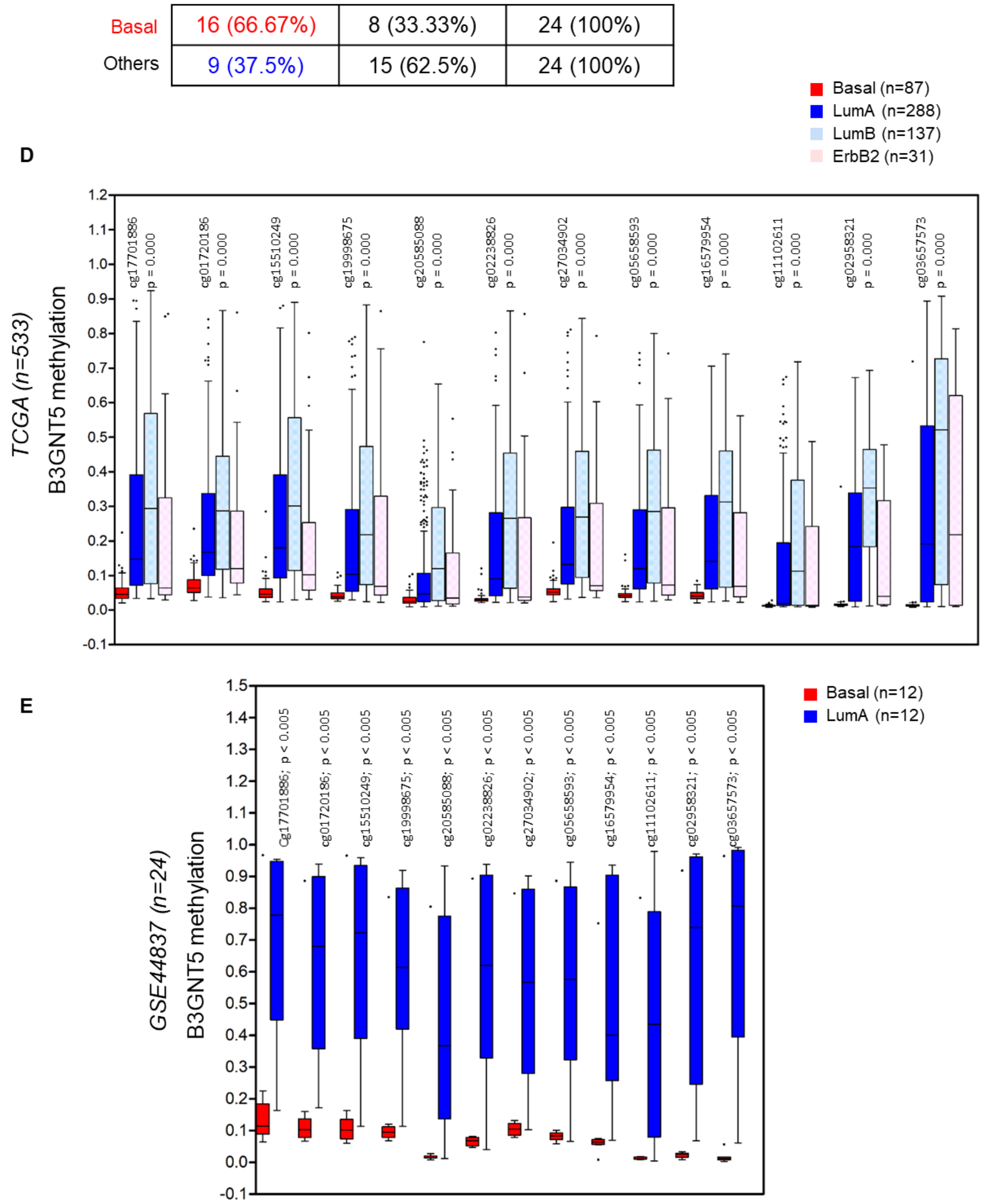

F

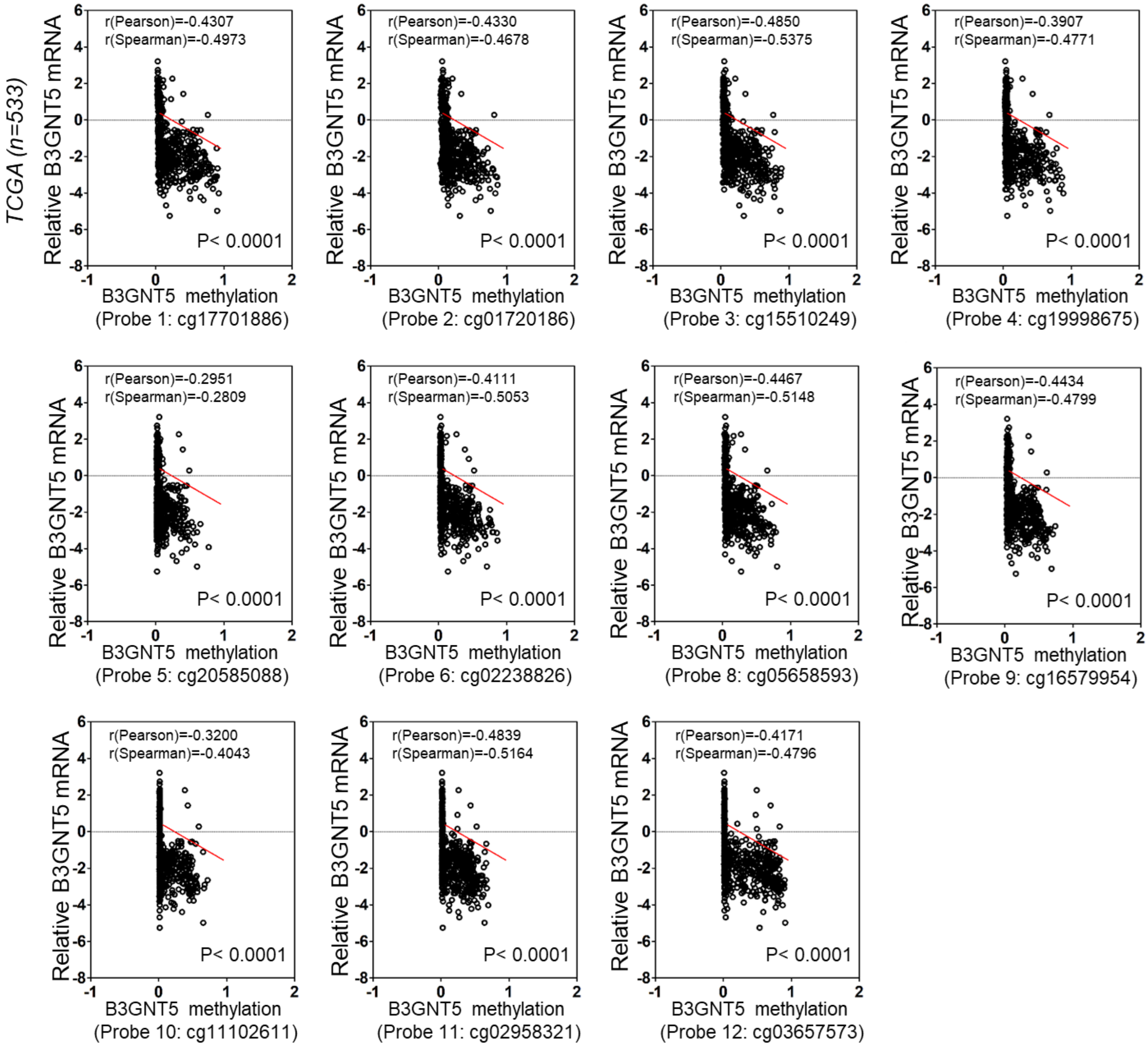
TCGA (n=533)
Relative B3GNT5 mRNA
r(Pearson)=-0.4307
r(Spearman)=-0.4973
P< 0.0001
B3GNT5 methylation
(Probe 1: cg17701886)
r(Pearson)=-0.4330
r(Spearman)=-0.4678
P< 0.0001
B3GNT5 methylation
(Probe 2: cg01720186)
r(Pearson)=-0.4850
r(Spearman)=-0.5375
P< 0.0001
B3GNT5 methylation
(Probe 3: cg15510249)
r(Pearson)=-0.3907
r(Spearman)=-0.4771
P< 0.0001
B3GNT5 methylation
(Probe 4: cg19998675)
r(Pearson)=-0.2951
r(Spearman)=-0.2809
P< 0.0001
B3GNT5 methylation
(Probe 5: cg20585088)
r(Pearson)=-0.4111
r(Spearman)=-0.5053
P< 0.0001
B3GNT5 methylation
(Probe 6: cg02238826)
r(Pearson)=-0.4467
r(Spearman)=-0.5148
P< 0.0001
B3GNT5 methylation
(Probe 8: cg05658593)
r(Pearson)=-0.4434
r(Spearman)=-0.4799
P< 0.0001
B3GNT5 methylation
(Probe 9: cg16579954)
r(Pearson)=-0.3200
r(Spearman)=-0.4043
P< 0.0001
B3GNT5 methylation
(Probe 10: cg11102611)
r(Pearson)=-0.4839
r(Spearman)=-0.5164
P< 0.0001
B3GNT5 methylation
(Probe 11: cg02958321)
r(Pearson)=-0.4171
r(Spearman)=-0.4796
P< 0.0001
B3GNT5 methylation
(Probe 12: cg03657573)

G

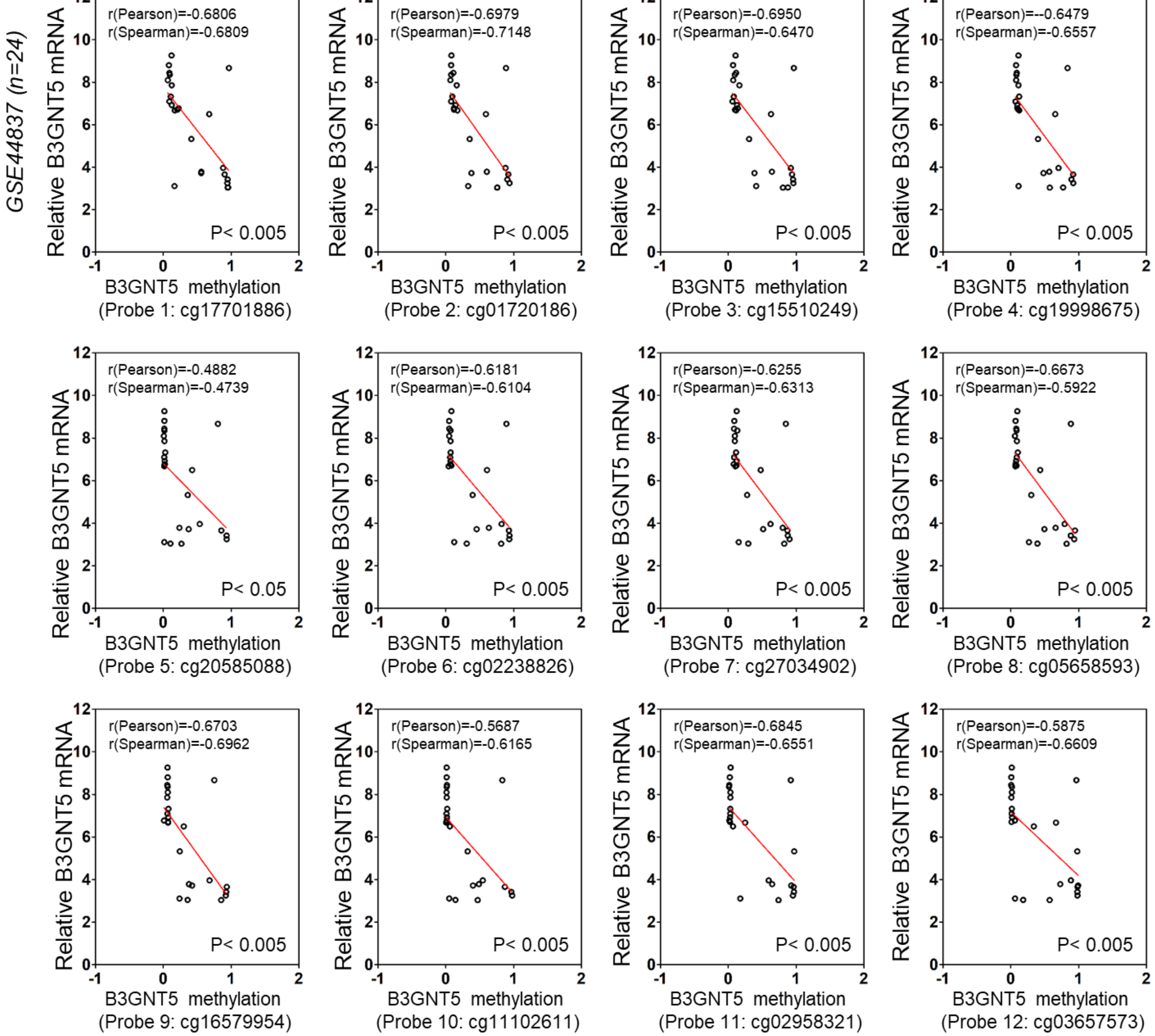
GSE44837 (n=24)
Relative B3GNT5 mRNA
r(Pearson)=-0.6806
r(Spearman)=-0.6809
P< 0.005
B3GNT5 methylation
(Probe 1: cg17701886)
r(Pearson)=-0.6979
r(Spearman)=-0.7148
P< 0.005
(Probe 2: cg01720186)
r(Pearson)=-0.6950
r(Spearman)=-0.6470
P< 0.005
(Probe 3: cg15510249)
r(Pearson)=--0.6479
r(Spearman)=-0.6557
P< 0.005
(Probe 4: cg19998675)
r(Pearson)=-0.4882
r(Spearman)=-0.4739
P< 0.05
(Probe 5: cg20585088)
r(Pearson)=-0.6181
r(Spearman)=-0.6104
P< 0.005
(Probe 6: cg02238826)
r(Pearson)=-0.6255
r(Spearman)=-0.6313
P< 0.005
(Probe 7: cg27034902)
r(Pearson)=-0.6673
r(Spearman)=-0.5922
P< 0.005
(Probe 8: cg05658593)
r(Pearson)=-0.6703
r(Spearman)=-0.6962
P< 0.005
(Probe 9: cg16579954)
r(Pearson)=-0.5687
r(Spearman)=-0.6165
P< 0.005
(Probe 10: cg11102611)
r(Pearson)=-0.6845
r(Spearman)=-0.6551
P< 0.005
(Probe 11: cg02958321)
r(Pearson)=-0.5875
r(Spearman)=-0.6609
P< 0.005
(Probe 12: cg03657573)

H

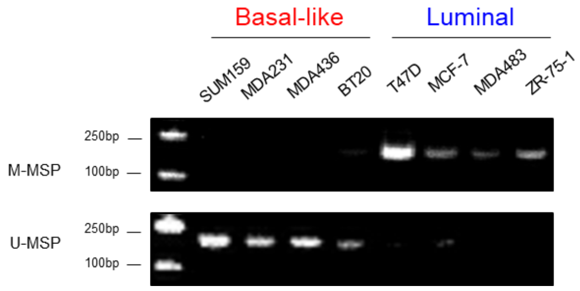
Basal-like
Luminal
SUM159
MDA231
MDA436
BT20
T47D
MCF-7
MDA483
ZR-75-1
M-MSP
U-MSP
250bp
100bp

Figure S3

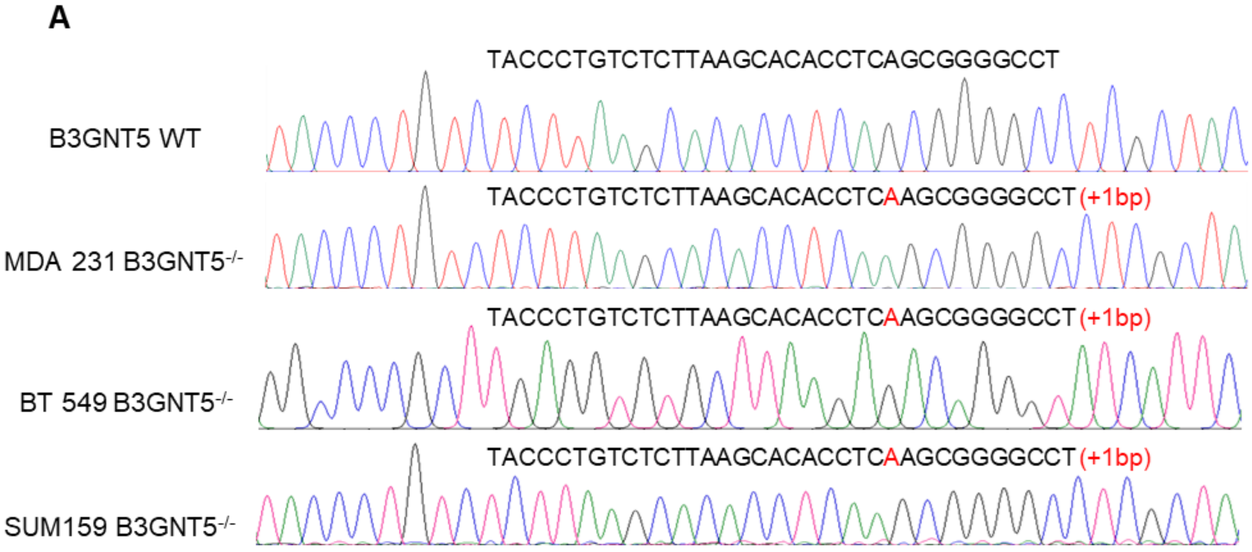
A
TACCCTGTCTCTTAAGCACACCTCAGCGGGGCCT
B3GNT5 WT
TACCCTGTCTCTTAAGCACACCTCAAGCGGGGCCT (+1bp)
MDA 231 B3GNT5-/-
TACCCTGTCTCTTAAGCACACCTCAAGCGGGGCCT (+1bp)
BT 549 B3GNT5-/-
TACCCTGTCTCTTAAGCACACCTCAAGCGGGGCCT (+1bp)
SUM159 B3GNT5-/-

Figure S4

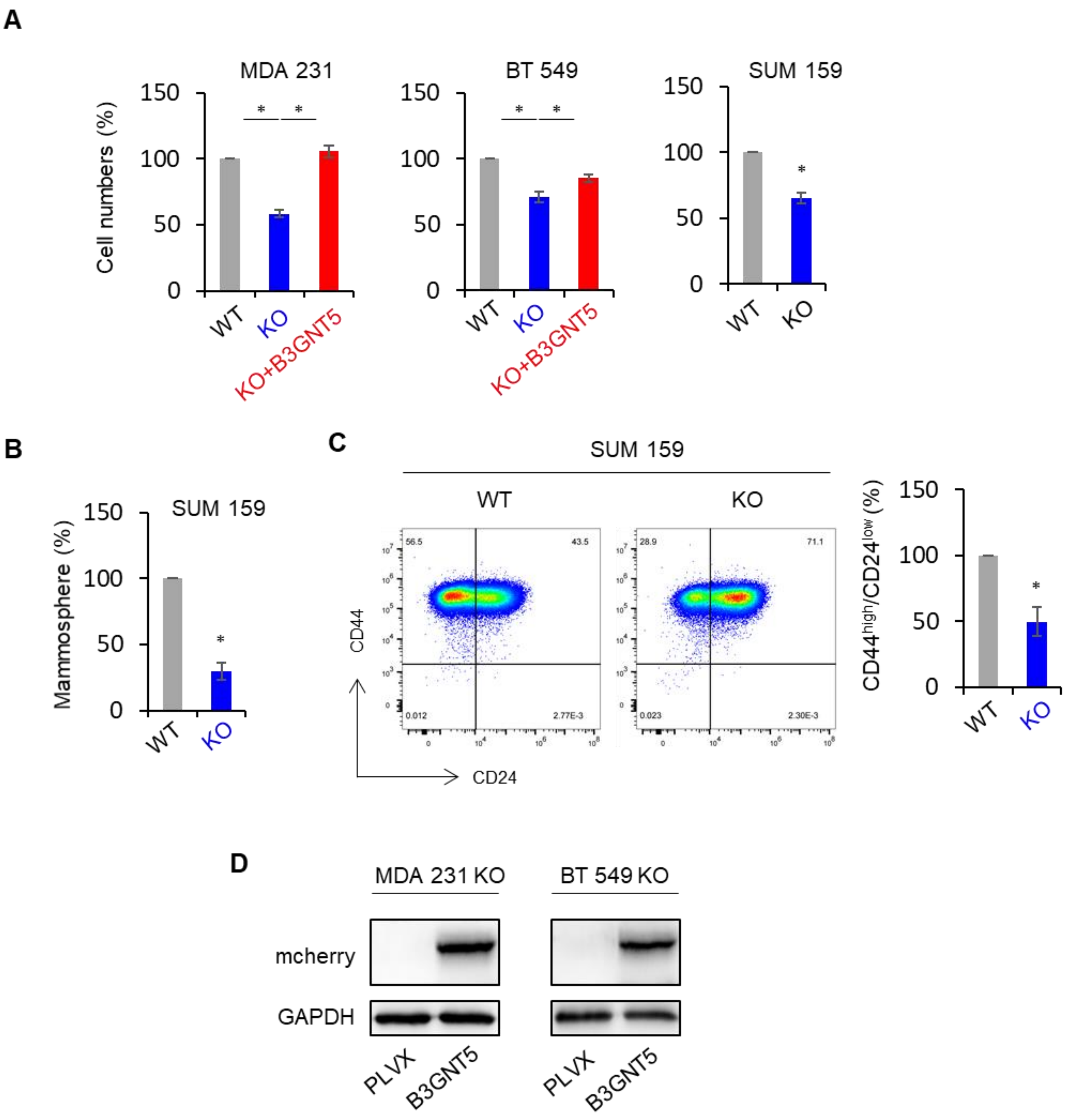

E

MDA 231 KO

Number of cells seeded	Reconstitution efficiency	
	mCherry Low	mCherry High
5	5/48	15/48
10	13/48	31/48
20	20/48	39/48
40	22/24	24/24
Stem cell frequency	1/30.5	1/11.5
(95% interval)	(1/39.3-1/23.8)	(1/14.1 -1/9.37)
%	0.0327	0.0869
p-value	<0.05	

BT 549 KO

Number of cells seeded	Reconstitution efficiency	
	mCherry Low	mCherry High
5	3/20	4/20
10	5/20	8/20
20	9/20	13/20
40	15/20	20/20
Stem cell frequency	1/ 31.9	1/ 17.0
(95% interval)	(1/ 45.4 -1/ 22.5)	(1/ 23.2 -1/ 12.5)
%	0.0313	0.0588
p-value	<0.05	

Figure S5

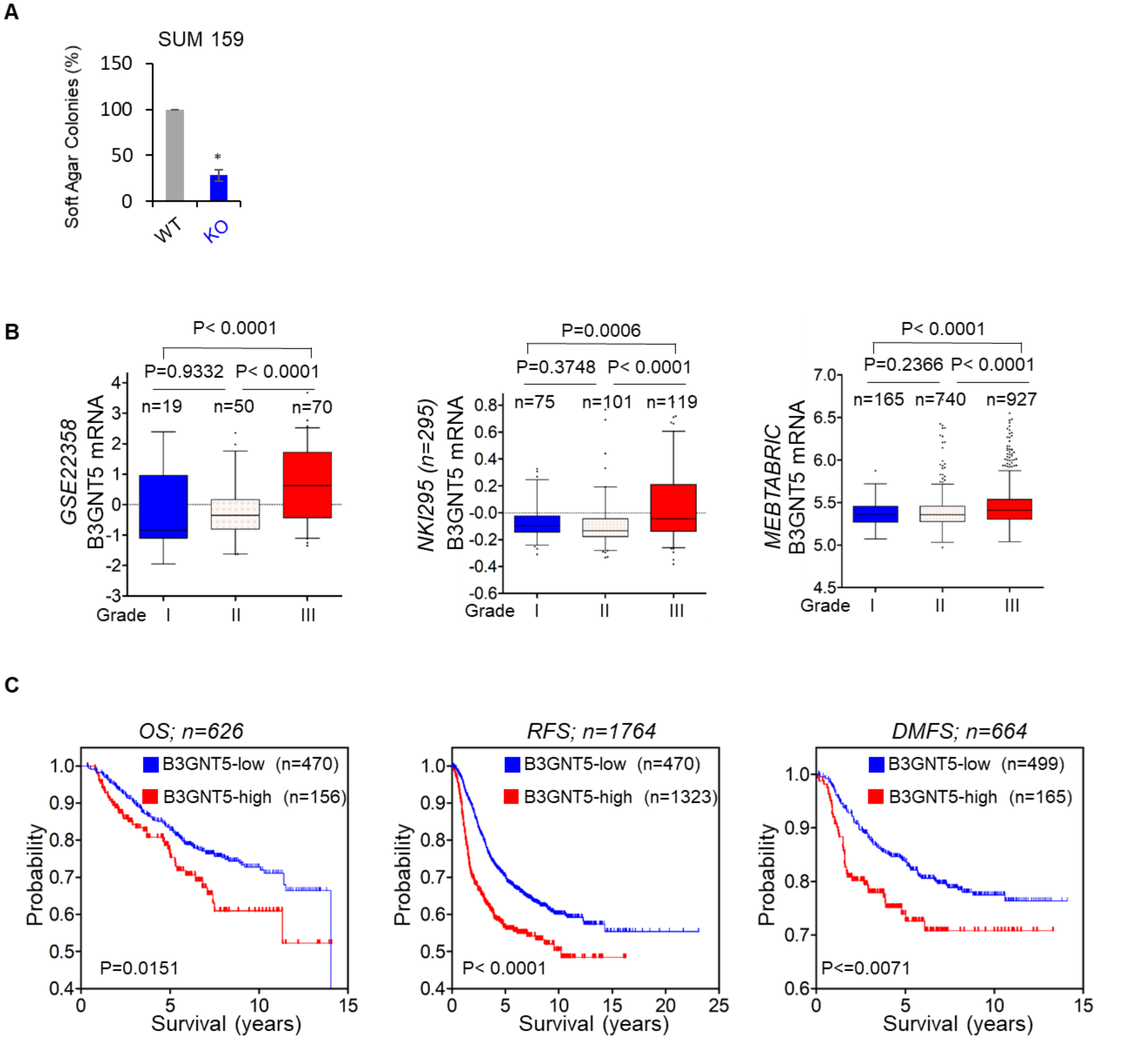

A
SUM 159
Soft Agar Colonies (%)
150
100
50
0
*
WT
KO
B
P< 0.0001
P=0.9332
P< 0.0001
n=19
n=50
n=70
GSE22358
B3GNT5 mRNA
Grade
I
II
III
P=0.0006
P=0.3748
P< 0.0001
n=75
n=101
n=119
NKI295 (n=295)
B3GNT5 mRNA
Grade
I
II
III
P< 0.0001
P=0.2366
P< 0.0001
n=165
n=740
n=927
MEBTABRIC
B3GNT5 mRNA
Grade
I
II
III
C
OS; n=626
B3GNT5-low (n=470)
B3GNT5-high (n=156)
P=0.0151
Probability
Survival (years)
RFS; n=1764
B3GNT5-low (n=470)
B3GNT5-high (n=1323)
P< 0.0001
Probability
Survival (years)
DMFS; n=664
B3GNT5-low (n=499)
B3GNT5-high (n=165)
P<=0.0071
Probability
Survival (years)

Figure S6

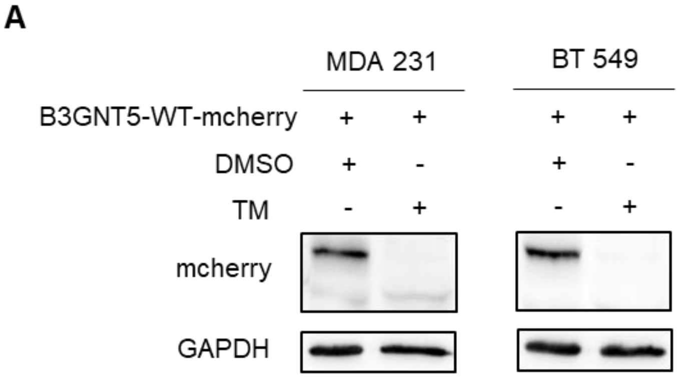
A
MDA 231
BT 549
B3GNT5-WT-mcherry
DMSO
TM
mcherry
GAPDH

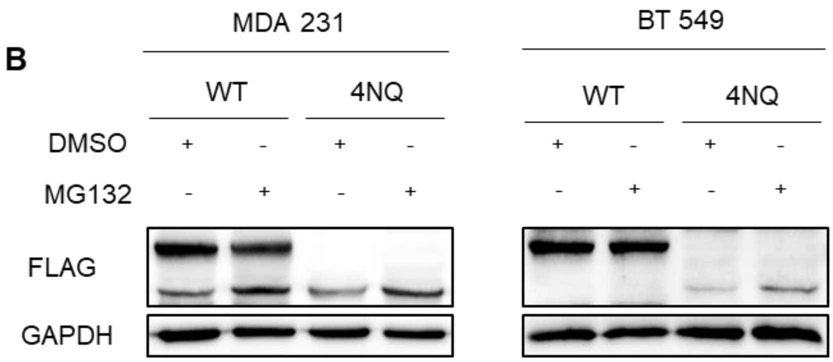
B
MDA 231
BT 549
WT
4NQ
WT
4NQ
DMSO
MG132
FLAG
GAPDH
